# Quantitative MRI of rostral spinal cord and brain regions is predictive of functional recovery in acute spinal cord injury

**DOI:** 10.1016/j.nicl.2018.08.026

**Published:** 2018-08-19

**Authors:** Maryam Seif, Armin Curt, Alan J. Thompson, Patrick Grabher, Nikolaus Weiskopf, Patrick Freund

**Affiliations:** a Spinal Cord Injury Center Balgrist, University of Zurich, Switzerland; b Department of Neurophysics, Max Planck Institute for Human Cognitive and Brain Sciences, Leipzig, Germany; c Department of Brain Repair and Rehabilitation, UCL Institute of Neurology, London, UK; d Wellcome Trust Centre for Neuroimaging, UCL Institute of Neurology, London, UK

**Keywords:** SCI, spinal cord injury, MT, magnetization transfer, R1, longitudinal relaxation rate, R2*, effective transverse relaxation rate, PD*, effective proton density, VBM, voxel based morphometry, VBCT, voxel based cortical thickness, VBQ, voxel based quantification, ROI, region of interest, MPM, multi-parameter mapping, APW, anterior posterior width, LRW, left right width, SCA, spinal cord area, ISNCSCI, international standards for the neurological classification of spinal cord injury, SCIM, spinal cord independence measure, Spinal cord injury, Quantitative neuroimaging, Acute micro-structural changes, Brain and spinal cord atrophy, Voxel-based morphometry and quantification

## Abstract

**Objective:**

To reveal the immediate extent of trauma-induced neurodegenerative changes rostral to the level of lesion and determine the predictive clinical value of quantitative MRI (qMRI) following acute spinal cord injury (SCI).

**Methods:**

Twenty-four acute SCI patients and 23 healthy controls underwent a high-resolution T1-weighted protocol. Eighteen of those patients and 20 of controls additionally underwent a multi-parameter mapping (MPM) MRI protocol sensitive to the content of tissue structure, including myelin and iron. Patients were examined clinically at baseline, 2, 6, 12, and 24 months post-SCI. We assessed volume and microstructural changes in the spinal cord and brain using T1-weighted MRI, magnetization transfer (MT), longitudinal relaxation rate (R1), and effective transverse relaxation rate (R2*) maps. Regression analysis determined associations between acute qMRI parameters and recovery.

**Results:**

At baseline, cord area and its anterior-posterior width were decreased in patients, whereas MT, R1, and R2* parameters remained unchanged in the cord. Within the cerebellum, volume decrease was paralleled by increases of MT and R2* parameters. Early grey matter changes were observed within the primary motor cortex and limbic system. Importantly, early volume and microstructural changes of the cord and cerebellum predicted functional recovery following injury.

**Conclusions:**

Neurodegenerative changes rostral to the level of lesion occur early in SCI, with varying temporal and spatial dynamics. Early qMRI markers of spinal cord and cerebellum are predictive of functional recovery. These neuroimaging biomarkers may supplement clinical assessments and provide insights into the potential of therapeutic interventions to enhance neural plasticity.

## Highlights

• Neurodegenerative changes rostral to the level of lesion occur early in SCI, with varying temporal and spatial dynamics.
• Early quantitative MRI markers of spinal cord and cerebellum are predictive of functional recovery.
• Quantitative MRI holds potential to supplement clinical assessment and provides insights into the therapeutic interventions.

## 1 Introduction

Spinal cord injury (SCI) is a devastating neurological disorder that leads to immediate sensorimotor and autonomic dysfunction below the lesion level (Freund et al., 2013; Grabher et al., 2015). SCI patients show limited clinical recovery and most patients are left permanently paralysed with significant degrees of impairment (Dietz and Fouad, 2014). Currently there is no cure for paralysis. Intensive neurorehabilitation fosters functional recovery within the first months after SCI (Gassert and Dietz, 2018) which is accompanied by time dependent neurodegenerative changes. A cascade of secondary neurodegenerative processes accompanies the recovery in SCI (Freund et al., 2013; Grabher et al., 2015; Park et al., 2004; Tator and Fehlings, 1991; Ziegler et al., 2018, Seif et al., 2018). Thus, understanding the interplay between neurodegenerative and reorganizational changes at the spinal and brain level during recovery would enable the development of evidence based rehabilitation therapy (Villiger et al., 2015).

Apart from early volumetric changes (atrophy) (Hou et al., 2014), it remains unclear whether microstructural changes including myelin and iron accumulation due to myelin breakdown (Sauerbeck et al., 2013) parallel the volumetric changes within the same regions and beyond, early after injury. Additionally, there is still limited knowledge on how the magnitude of early macro- and microstructural changes relate to functional recovery following SCI.

Various advanced quantitative magnetic resonance imaging (qMRI) methods have already shown significant potential to provide quantitative measures of neurodegenerative changes in both brain and the spinal cord (Calabrese et al., 2018; Laule et al., 2006; Martin et al., 2017; Schmierer et al., 2018, Schmierer et al., 2007b, Schmierer et al., 2004; Villiger et al., 2015). Multi-parameter mapping (MPM) provides quantitative maps which are indirectly sensitive to the content of tissue structure including myelin and iron (Dick et al., 2012; Draganski et al., 2011; Helms et al., 2008; Sereno et al., 2013; Weiskopf et al., 2013).

Previous studies applied qMRI in SCI and have shown early trauma-induced volumetric decreases (i.e. atrophy) (Hou et al., 2014) as well as task-related increases in brain activity (i.e. reorganization) during upper limb recovery (Jurkiewicz et al., 2007) in the primary motor cortex (M1) occurring within the first months following injury. Moreover, the cerebellar circuitry undergoes significant alterations after SCI which affects ascending spinocerebellar pathways (Visavadiya and Springer, 2016) and has been related to increases in the intensity of neuropathic pain (Grabher et al., 2015). Interestingly, the limbic system is prone to structural changes as well, some of which relate to clinical impairment (Grabher et al., 2015; Jutzeler et al., 2016). These studies indicate that neurodegenerative and reorganizational processes occurring early and in parallel after injury. However, relatively little is known as to how early after injury remote (micro-) structural changes become evident and if they are predictive of functional recovery. Our study therefore aimed to identify neurodegeneration based on macro- and microstructural MRI parameters above the level of injury (in the cervical cord, cerebellum, M1 and limbic system) within a few weeks after injury and to assess the predictive validity of early changes in MRI parameters for functional recovery over a period of two years following SCI.

## 2 Material and methods

### 2.1 Participants

The local cantonal ethics committee of Zurich approved the study (EK-2010-0271), and informed written consent was obtained from each subject before participation. The longitudinal aspect of neurodegeneration following spinal cord injury have been previously reported in a subset of participants included in the present study (Freund et al., 2013; Ziegler et al., 2018; Seif et al., 2018).

Twenty-four acute SCI patients (mean age = 49.7 ± 19.8 years, 5 female) with mean post-SCI period of 45.6 ± 20.7 days and 23 healthy controls (age = 35.9 ± 10.9 years, 10 female) were enrolled in this study at the University Hospital Balgrist between July 2010 and July 2014 (Table 1). The exclusion criteria were: time since injury >2 months, pregnancy, head or brain lesions associated with spinal cord injury, pre-existing neurological and medical disorders leading to functional impairments, mental disorder, or contraindications to MRI. Patients were clinically examined and scored on the international standards for the neurological classification of spinal cord injury (ISNCSCI) protocol (Kirshblum et al., 2011). Within this protocol, the muscle strength is assessed (on a scale form 0 to 5, 0 indicates no voluntary control while 5 indicates full strength) on key muscles in the upper and lower extremity. To assess the sensory integrity based on the ISNCSCI, the perception to light touch and pinprick is scored (0 = no sensation, 1 = abnormal sensation, 2 = normal sensation) on all the dermatomes. Functional independence was assessed by the spinal cord independence measure (SCIM) (Anderson et al., 2008) which scores the ability of the individual to perform daily activities (i.e. self-care, respiration and sphincter management and mobility. All clinical assessments were applied within predefined five-time points: baseline (about 46 day's post-SCI), 2 months, 6 months, 12 months, and 24 months after injury.

**Table 1:** Clinical information of 24 patients at baseline; MVA: Motor vehicle accident, AIS = ASIA impairment scale.

ID	Gender	Age at Baseline measure (years)	AIS grade at Baseline	Initial site of impairment (motor/sensory)	Type of injury
1	m	18	A	C4/C4	Fall
2	f	72	A	T11/T11	Ischemia
3	m	30	A	C7/C7	Fall
4	m	28	A	C7/C5	Fall
5	m	42	A	C5/C6	Fall
6	m	69	A	T7/T7	MVA
7	m	70	B	C7/C8	Fall
8	m	20	B	C5/C5	MVA
9	m	30	B	C7/C8	MVA
10	m	29	B	L2/L3	Fall
11	m	51	B	C7/C5	MVA
12	m	22	C	C6/C8	Fall
13	f	68	C	T10/T10	Ischemia
14	m	71	C	T10/T10	Ischemia
15	f	53	C	C4/C4	Fall
16	f	46	D	T8/T8	Ischemia
17	m	73	D	S2/S2	Fall
18	m	77	D	T10/T10	Ischemia
19	m	43	D	L3/L3	Fall
20	m	52	D	T9/T9	Fall
21	m	47	D	C4/C4	MVA
22	m	67	D	C3/C3	Fall
23	f	71	D	T4/T4	Ischemia
24	m	32	D	T3/T3	Fall

### 2.2 MRI measurements

All subjects underwent a high-resolution T1-weighted 3D Magnetization Prepared Rapid Acquisition Gradient-Echo (MPRAGE) sequence (whole-brain extending to the cervical C5 level) with following parameters: field of view (FOV) = 224 × 256 mm^2^, matrix-size = 224 × 256, repetition time (TR) = 2420 milliseconds, echo time (TE) = 4.18 milliseconds, Bandwidth = 150 Hz/pixel, and resolution = 1x1x1 mm^3^ using a 3 T Magnetom Skyra^Fit^ and Verio MRI scanner (Siemens Healthcare, Erlangen, Germany) combined with a 16-channel receive radio-frequency (RF) head-neck coil for assessing both the cervical spinal cord and brain.

To assess microstructural changes associated with quantitative MR parameters, 18 patients and 20 controls underwent the MPM MRI protocol (Helms et al., 2008; Weiskopf et al., 2013) which composed of three different 3D multi-echo fast low-angle shot (FLASH) gradient-echo sequences, designed to provide MR parameter measures of longitudinal relaxation rate (R1 = 1/T1), effective proton density (PD*), magnetization transfer saturation (MT) and effective transverse relaxation rate (R2* = 1/T2*) with 1 mm isotropic resolution and FOV = 240 × 256 mm^2^ (matrix-size = 5240 × 256) with 176 partitions in a total scan time of 23 min and applying parallel imaging in the phase-encoding direction using a generalized auto-calibration partially parallel acquisition algorithm (GRAPPA) factor 2 × 2. The readout bandwidth was 480 Hz/pixel. The following parameters were: TR = 25 milliseconds, flip-angle = 23° and 4° for T1-weighted images and PD-weighted images, respectively. TR = 37 milliseconds, flip-angle = 9° for MT-weighted images, six echoes between 2.46 and 14.78 milliseconds for the MT-weighted acquisitions with two additional echoes at 17.22 milliseconds and 19.68 milliseconds for the T1-weighted and the PD-weighted acquisitions. Note, the MPM protocol was installed after the commencement of the study and therefore the first participants (5 healthy controls and 6 patients) underwent only the MPRAGE sequence.

### 2.3 Image analysis

#### 2.3.1 Cervical cord analysis

The cross-sectional spinal cord area (SCA) was calculated at C2/C3 level applying a semi-automatic active surface model on the T1-MPRAGE images using Jim 7.0 software (Xinapse systems, Aldwincle, UK). Further, we applied an ellipse fitting to calculate the anterior-posterior width (APW) and left-right width (LRW) (Grabher et al., 2015) using an in-house MATLAB (R2013b) script. Next, we defined the cord volume within the MT map followed by the same ellipse fitting procedure using an in-house MATLAB script based on nearest neighbour region growing algorithm. The ROI for the spinal cord was superimposed on the R1 maps and used to extract the mean quantitative parameters from the MT and R1 maps (Grabher et al., 2015).

#### 2.3.2 Whole brain analysis

Voxel based morphometry (VBM) and voxel based cortical thickness (VBCT) methods were applied to T1w-MPRAGE images for estimation of grey matter (GM) and white matter (WM) changes (Ashburner and Friston, 2000) and investigate the cortical thickness changes of the whole-brain (Hutton et al., 2008), respectively.

We segmented T1w-MPRAGE images into GM, WM, and cerebrospinal fluid (CSF) with unified segmentation (Ashburner and Friston, 2005) in VBM analysis. For each subject, this procedure produced three images in the same space as the original anatomical image, in which each voxel was assigned a probability of being GM, WM, or CSF. Next, the GM and WM segments were spatially normalized into standard MNI space, with a diffeomorphic Anatomical Registration using Exponentiated Lie algebra (Dartel) algorithm (Ashburner, 2007). The grey matter and white matter maps were subsequently modulated by the Jacobian determinants of the deformations (Good et al., 2001). Finally modulated probability maps were smoothed with an isotropic Gaussian kernel of 3 mm full width at half maximum (FWHM). The total intracranial volume (TIV) was calculated as sum of grey matter, white matter, and CSF volumes (Ridgway et al., 2011).

Moreover, we applied voxel based quantification (VBQ) (Draganski et al., 2011; Weiskopf et al., 2013) on MPM images to assess microstructural changes which includes the indirect measure of myelin (MT & R1) and iron content (R2*) (Langkammer et al., 2010; Weiskopf et al., 2014) using general linear models within the framework of the SPM12 (University College London, London, UK). MT-weighted, PD-weighted, and T1-weighted images acquired from MPM protocol were used to calculate quantitative parameter maps of MT, R1, and R2*. To estimate the inhomogeneity of the RF transmit filed, we used unified segmentation based correction (UNICORT) (Weiskopf et al., 2011) on R1 maps. We also segmented the MT images to GM, WM and CSF using unified segmentation method (Ashburner and Friston, 2005). The Dartel algorithm (Ashburner, 2007) was applied for the transformation to MNI space, maps were warped to the MNI space with the participant specific flow fields from the MT maps and smoothed with an isotropic Gaussian kernel filter with 3 mm full-width at half maximum (FWHM). The VBQ approach was used for this normalization process to minimize partial volume effects and for relative contribution of GM and WM accounting to the respective voxel signal.

#### 2.3.3 Region of interest (ROI)

The ROI approach applied in brain analysis, based on the previous studies (Freund et al., 2011, Freund et al., 2013; Grabher et al., 2015; Jutzeler et al., 2016) comprised of bilateral M1 and S1 cortices (precentral and postcentral gyrus) respectively, cerebellum, and thalamus extracted from SPM Anatomy toolbox (Eickhoff et al., 2007). Moreover, to increase sensitivity of changes within the leg area of M1, a 10 mm sphere was centred on bilateral x = 6, y = −28, z = 60 based on previous reports (Freund et al., 2011).

### 2.4 Statistical analysis

To investigate macrostructural changes (cervical cord area, APW and LRW) and microstructural changes (MT, R1) in the cord between groups, two-sample *t*-test in Stata (Stata Corp 13.0, College Station, TX) was used. We applied linear regression models to explore relationships between early structural changes of the cord area and functional recovery (Lower extremity motor score (LEMS), light touch, pinprick, and SCIM scores) over time (patients only) adjusted for clinical baseline status, age, and gender.

General linear models were used to assess brain volume changes in GM and WM, and the microstructure in defined ROIs at the group level. Age and TIV were included as covariates of no interest. Uncorrected voxel threshold of *p* = .001 was initially considered in statistical parametric maps. To account for multiple comparisons, we applied theory of Gaussian random fields and only clusters surviving a corrected cluster threshold of *p* = .05 (family wise error corrected (FWE) based on Gaussian random field theory) were reported as significant (Friston et al., 1994). One tailed *t*-tests with a significant threshold of *p* < .05 were used in each voxel of interest to test for decreases in patients and to compare the microstructural changes between controls and patients. To ensure that each voxel was analysed only once, either in GM or WM segments, explicit masks for each subspace were generated by assigning each voxel with a probability >20% to the tissue class with the highest probability (Grabher et al., 2015). We explored associations between structural changes at baseline (46 days' post-SCI) and functional recovery (LEMS, light-touch, pinprick and SCIM scores) at 2 months, 6 months (short term), 12 months, and 24 months (long term), adjusting for potentially confounding effects of clinical baseline status, age, and gender. Only significant associations with p < .05 are reported.

## 3 Results

### 3.1 Spinal cord analysis

**Fig. 1:**
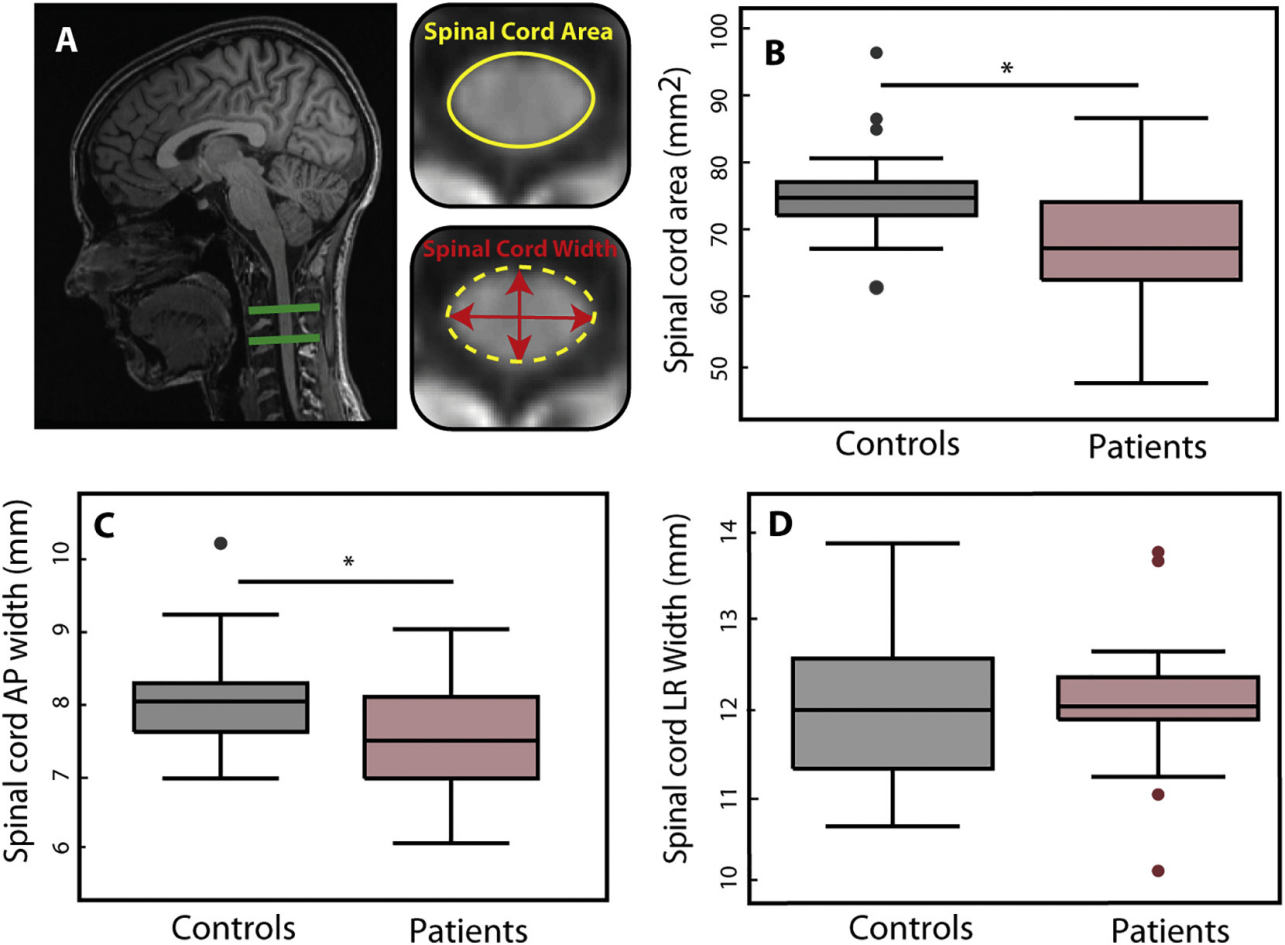
Macrostructural changes in the cervical cord A: Illustration of measures of cross sectional spinal cord area, left-right width (LRW) and anterior-posterior width (APW) at cervical level C2-C3. Box plot shows B: Reduced cross-sectional cord area (p = .004) C: Reduced APW of the spinal cord (p = .005) D: reduced LRW of the spinal cord. All parameters were determined at the C2/C3 level in patients compared to controls. Dots represent outliers that fall below Q1–1.5 × IQR or above Q3 + 1.5 × IQR (Q1: first quartile, Q3: third quartile, IQR = Q3-Q1: interquartile range).

At baseline the cervical cord area (*p* = .004 patients: SCA = 69.23±9.47 mm^2^, controls = 74.95±7.57 mm^2^) and its APW (*p* = .005, patients = 7.54±0.76 mm; controls = 8.12±0.67 mm) were already decreased in patients, while the LRW remained unchanged (*p* = .67, patients = 12.09±0.74 mm; controls = 12.00 ± 0.76) (Fig. 1). Microstructural measures of MT, R1 and R2* in the cervical cord were not significantly different in patients compared to controls.

### 3.2 Whole brain analysis in grey matter, white matter and cortical thickness

**Fig. 2:**
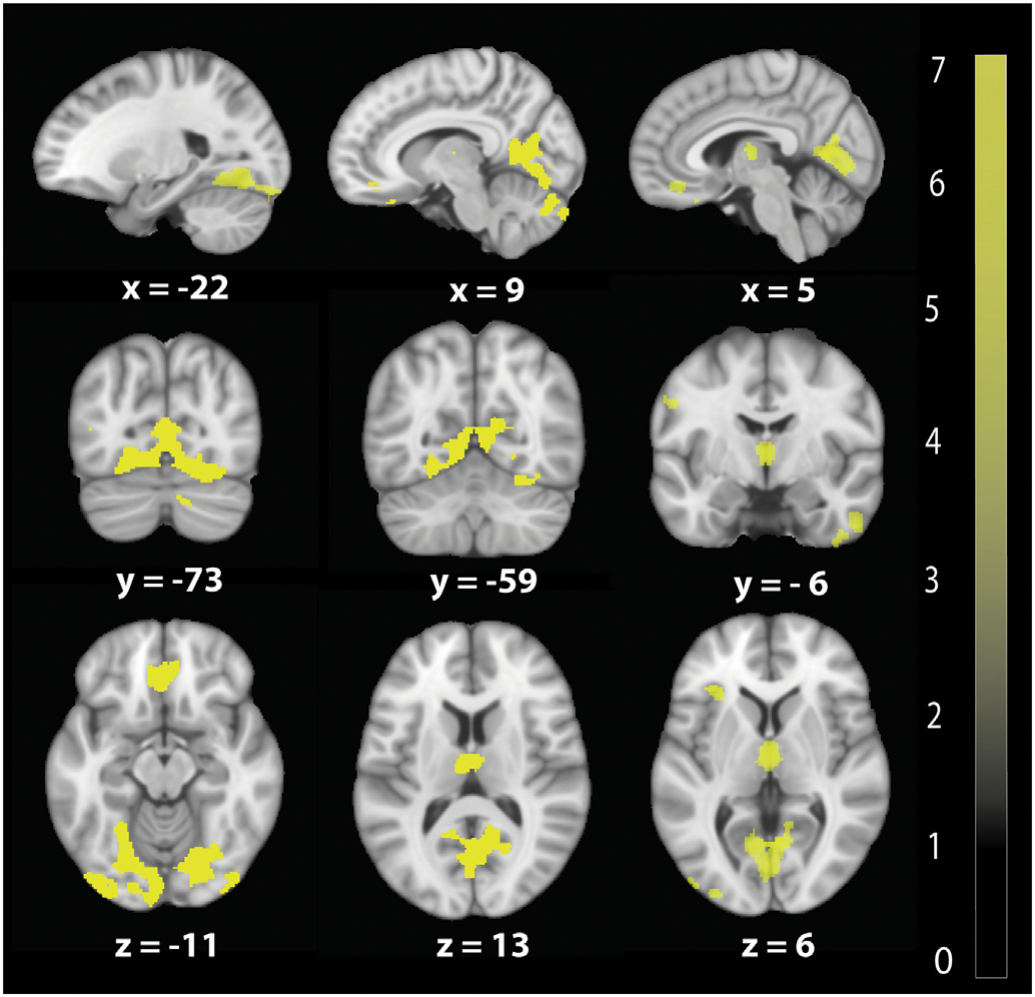
Baseline volumetric changes in brain revealed by voxel-based morphometry (VBM). Overlay of statistical parametric maps in grey matter shows volumetric decreases in bilateral thalamus, in bilateral lingual gyrus extending into the cerebellum, and in the left inferior frontal gyrus in patients compared to control. Overlay of statistical parametric maps are uncorrected *P* < .001, for illustrative purposes. The colour bar indicates the t score.

At baseline VBM analysis revealed significant GM volume decreases in a number of areas- the left anterior insula (z-score = 5.01, x = −35, y = 30, z = 5, *p* = .009, cluster extent (CE) = 743), the bilateral thalamus (z-score = 4.70, x = 0, y = −11, z = 6, *p* = .007, CE = 780), the bilateral anterior cingulate gyrus (z-score = 4.50, x = −2, y = 35, z = −15, *p* = .001, CE = 1203), and the right lingual gyrus extending into the right cerebellum and occipital gyrus (z-score = 5.83, x = 2, y = −62, z = 8, *p* < .0001, CE = 982) (Fig. 2). VBM of WM did not show changes in patients compared with controls.

**Fig. 3:**
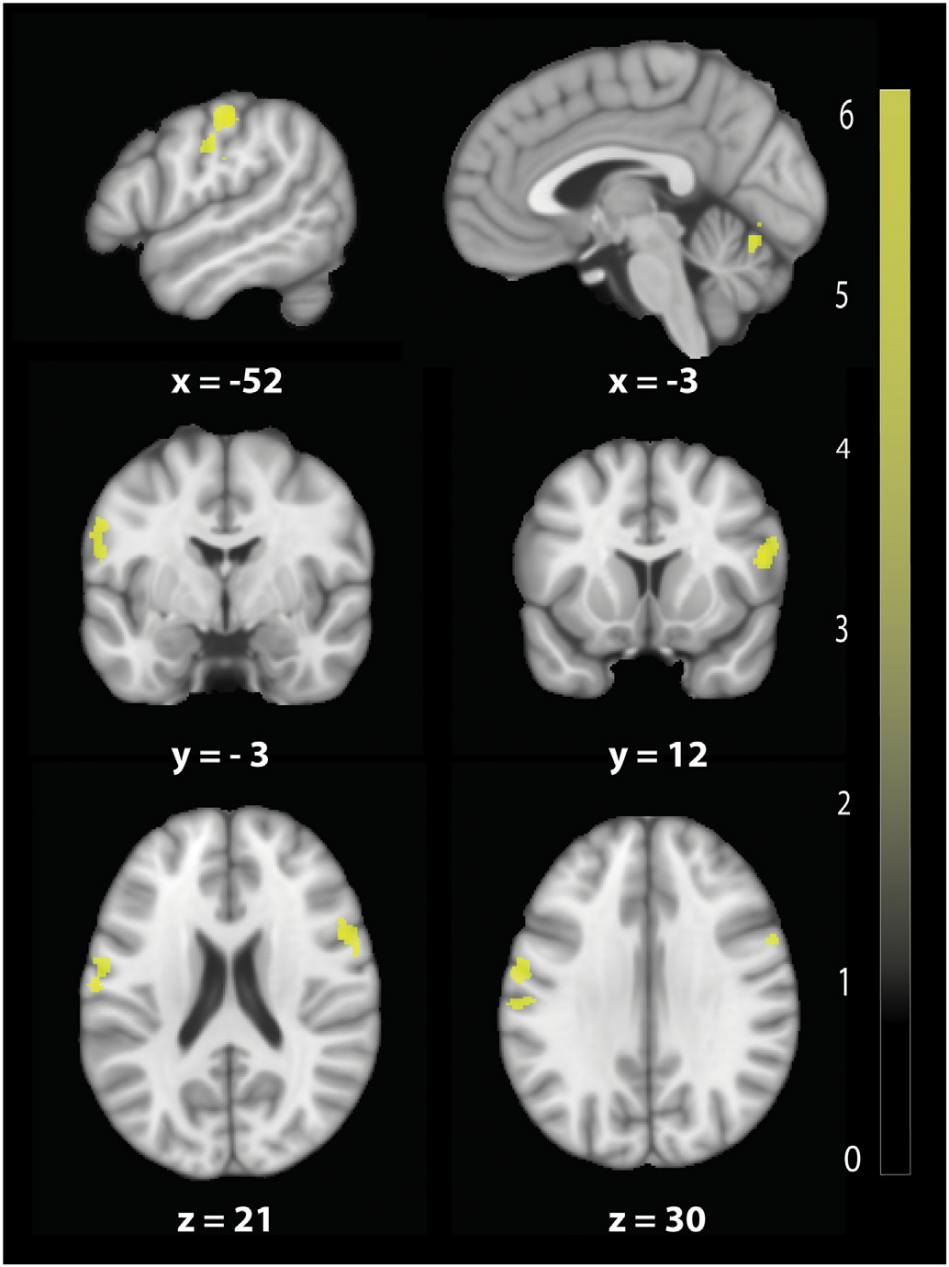
Baseline cortical thickness changes in bilateral M1/S1 and cerebellum in patients compared to healthy controls revealed by voxel based cortical thickness analysis (VBCT) (uncorrected p < .001, for illustrative purposes). The colour bar indicates the t score.

VBCT revealed reduced cortical thickness (Fig. 3.) in bilateral M1 (z-score = 4.79, x = 53, y = 11, z = 18, *p* = .039, CE = 412), in bilateral S1 (z-score = 4.84, x = −54, y = −23, z = 50, *p* = .001, CE = 858), and in right lingual gyrus extending into cerebellum (z-score = 4.06, x = 14, y = −80, z = −15, *p* = .01, CE = 572).

### 3.3 Region of interest (ROI) analysis in brain

**Fig. 4:**
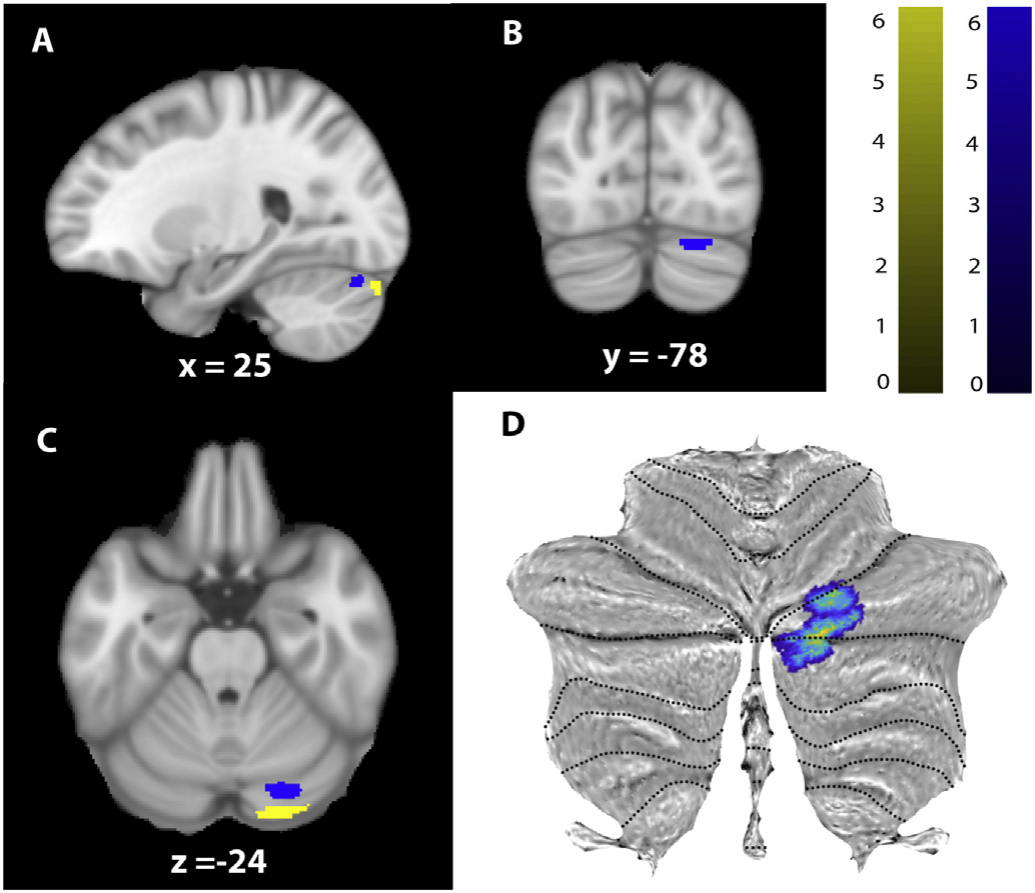
Microstructural changes in grey matter at baseline revealed by voxel-based quantification (VBQ). A, B, and C: Overlay of statistical parametric maps (uncorrected *p* < .001, for illustrative purposes) showing elevated MT in blue and increased effective transverse relaxation (R2* = 1/T2*) in yellow in patients compared to controls in the cerebellum. The colour bar indicates the t score. D: The cerebellar results are overlayed onto the flattened map of the cerebellum using SUIT toolbox (Diedrichsen et al., 2009).

The more sensitive ROI based approach revealed significant GM volume decreases in the right cerebellum (z-score = 4.68, x = 24, y = −74, z = −18, p < .0001, CE = 1088), in the area of M1 representing the left leg (z-score = 3.96, x = −53, y = −11, z = 32, *p* = .017, CE = 400), and in the right S1 (z-score = 4.07, x = 44, y = −17, z = 42, *p* = .013, CE = 434). VBCT showed cortical thinning in the bilateral cerebellum (GM) (z-score = 4.01, x = 12, y = −77, z = −15, *p* = .032, CE = 259) and in the area of M1 representing the left leg (z-score = 3.51, x = 5, y = −33, z = 63, p = .03, CE = 21) and in the left S1, in the same area of brain reported in whole brain analysis before, (z-score = 4.85, x = −54, x = −23, z = 50, p = .001, CE = 861). VBQ revealed increased MT in the right cerebellum (GM) (z-score = 5.58, x = 24, y = −80, z = −24, *p* = .009, CE = 98) and in cerebellar vermal lobules VI-VII (z-score = 4.11, x = 3, y = −74, y = −20, *p* = .033, CE = 71) as well as increased R2* in right cerebellum (z-score = 5.02, x = 24, y = −87, z = −26, *p* = .002, CE = 160) in patients compared to controls (Fig. 4). All these results are reported in Table 2.

**Table 2:** Results of whole brain and region of interest (ROI) analysis using voxel based morphometry (VBM), voxel based cortical thickness (VBCT), and voxel based quantification (VBQ) methods; LT: Light touch score, LEMS: Lower extremity score.

	Brain area	*Z*-score	*P*-value (FWE corrected)	Cluster extent	X (mm)	Y (mm)	Z (mm)
*Whole brain analysis*							
VBM (GM)	Left anterior insula	5.01	=0.009	743	−35	30	5
	Bilateral thalamus	4.70	=0.007	780	0	−11	6
	Bilateral anterior cingulate gyrus	4.50	=0.001	1203	−2	35	−15
	Bilateral lingual gyrus extending to cerebellum	5.83	=0.0001	982	2	−62	8
VBCT	Bilateral M1	4.79	=0.039	412	53	11	18
	Bilateral S1	4.84	=0.001	858	−54	−23	50
	Right lingual gyrus extending to Cerebellum	4.06	=0.01	572	14	−80	−15

*Region of interest (ROI) analysis in brain*							
VBM (GM)	Right cerebellum	4.68	=0.0001	1088	24	−74	−18
	M1 representing left leg area	3.96	=0.013	400	−53	−11	32
	Right S1	4.07	=0.032	434	44	−17	42
VBCT	Bilateral cerebellum	4.01	=0.032	259	12	−77	−15
	Right M1 representing right leg	3.51	=0.03	21	5	−33	63
	Left S1	4.85	=0.001	861	−54	−23	50
VBQ (MT, R2*)	Right Cerebellum (MT)	5.58	=0.009	98	24	−80	−24
	Cerebellar vermal lobules VI-VII (MT)	4.11	=0.033	71	3	−74	−20
	Right cerebellum (R2*)	5.02	=0.002	160	24	−87	−26

*Baseline predictors of outcome in brain*							
GM volume & LT score at 12 M	Left cerebellum	4.02	=0.03	326	−14	−93	−30
R2* & LEMS scores at 6 M	Right cerebellum	6.23	=0.002	158	14	−69	−57
R2* & LEMS scores at 12 M	Right cerebellum	5.30	=0.009	117	14	−71	−57

### 3.4 Baseline predictors of outcome

**Fig. 5:**
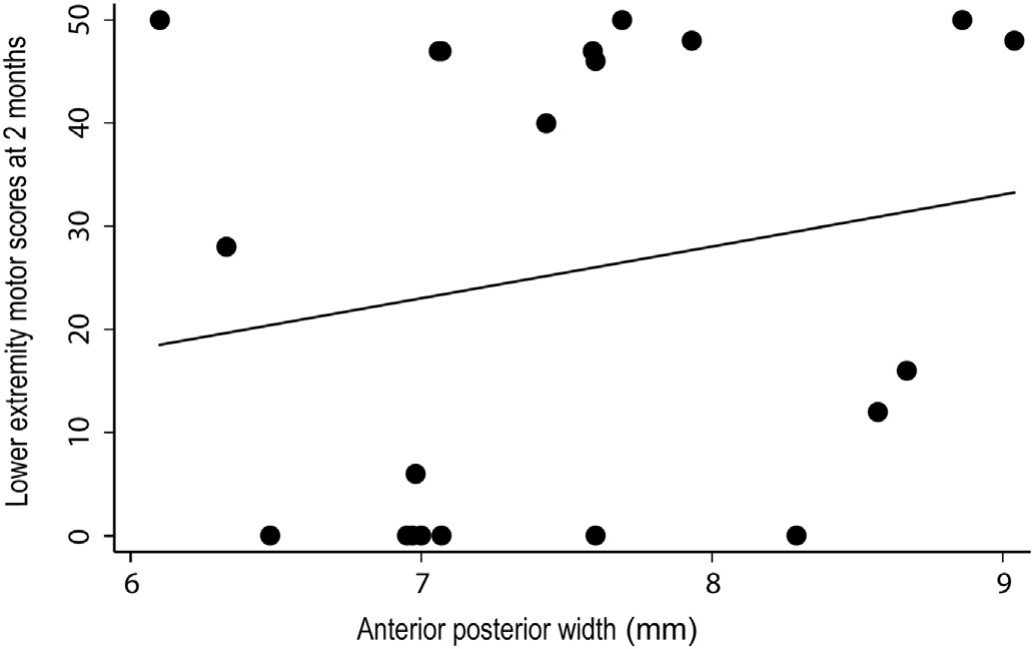
Correlation between anterior-posterior width and lower extremity motor scores at 2 months following injury (p = .03, r^2^ = 0.87) at the C2-C3 cord level.

**Fig. 6:**
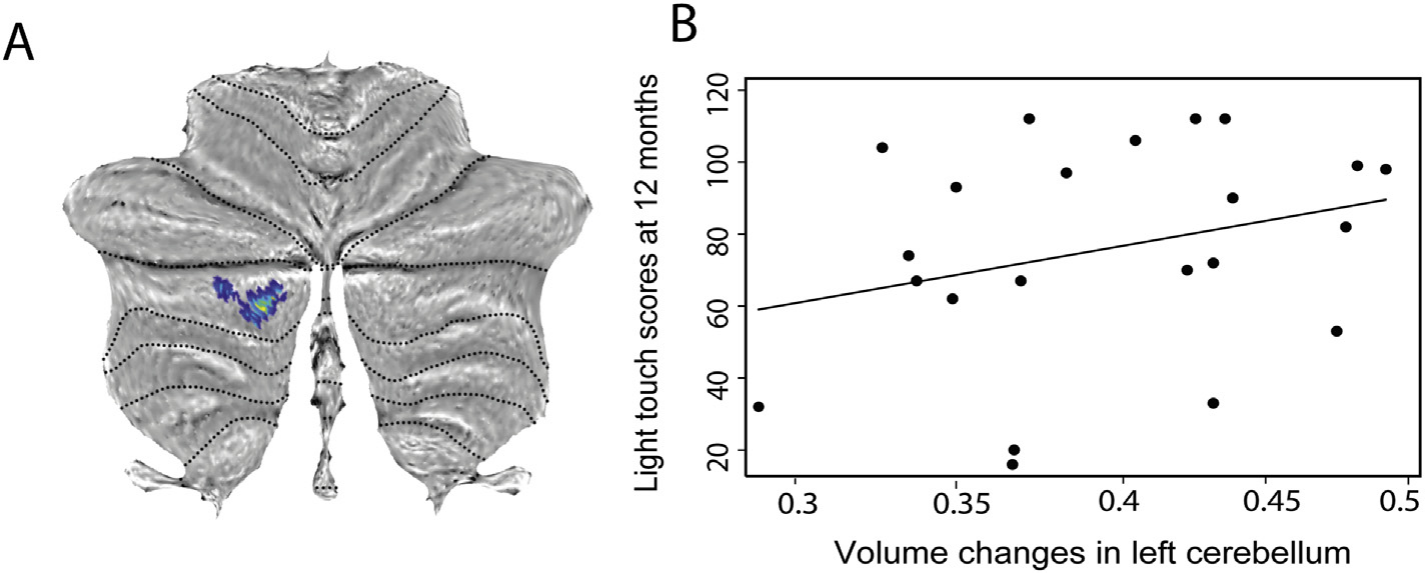
Correlation between atrophy in the cerebellum and sensory outcome (light touch score at 12 months). Overlay of statistical parametric maps on the flattened map of the cerebellum for demonstration purpose (Diedrichsen et al., 2009).

At the cord level, baseline APW of the cord area was associated with lower extremity motor scores at two months (p = .03, r^2^ = 0.87; [95% Confidence interval (CI): 0.55 to 10.3]) (Fig. 5). Baseline R1 of the cord was associated with pin-prick score at twelve months (*p* = .04, r^2^ = 0.71; [CI: -0.12 -0.002]). In the brain, GM volume decreases in the left cerebellum was associated with light touch scores at twelve months (z-score = 4.02, x = −14, y = −93, z = −30, *p* = .03, CE = 326) (Fig. 6). Baseline R2* in the right cerebellum was associated with lower extremity motor scores at 6 months (z-score = 6.23, x = 14, y = −69, z = −57, p = .002, CE = 158) and at 12 months (z-score = 5.3, x = 14, y = −71, z = −57, p = .009, CE = 117). No associations were evident between baseline qMRI parameters of the brain and clinical outcomes at 24 months. Similarly there were no significant correlations between the average time since injury and clinical recovery.

## 4 Discussion

In this study, we applied qMRI early after SCI and determined remote atrophy (i.e. volume decreases and cortical thickness changes) and microstructural changes sensitive to different tissue components including myelin and iron at the rostral cord and brain level. To our knowledge this is the first study to investigate the microstructural changes in acute SCI. The magnitude of trauma-induced structural changes at baseline predicted functional recovery during follow-up. These findings, in the context of previous reports (Freund et al., 2013; Grabher et al., 2015; Ziegler et al., 2018), allowed us to address two important questions: how soon following injury are the macro- and microstructural changes detectable and whether neurodegenerative changes at the earliest time points are associated with functional recovery.

Our previous longitudinal studies from a subgroup of the current patient cohort revealed that progressive changes in macroscopic and microstructural qMRI markers continue for at least two years post-trauma (Seif et al., 2018; Ziegler et al., 2018). While these progressive changes level off within the spinal cord, they showed sustained changes as a linear function of time in sensorimotor areas and other regions of the central nervous system. In this study, the cervical cord above the level of injury showed significant signs of atrophy at 46 days following SCI, while microstructural changes only become evident with a certain time lag (Grabher et al., 2015). As cord atrophy is representative of an accumulation of multiple disease processes, several possible mechanisms need to be considered. Immediately following trauma, a cascade of pathophysiological processes at the site of injury is initiated (Rossignol et al., 2007). The most prominent processes include damage to neurons and oligodendrocytes (Starkey and Schwab, 2012), the expression of neurotrophic factors from non-neuronal cells around neighboring degenerating axons (Lemon and Griffiths, 2005), growth-factor dysregulation (Bareyre and Schwab, 2003), vascular remodelling, and disruption of the blood–spinal cord barrier (Tator and Fehlings, 1991). The primary injury processes initiate a secondary response (Crowe et al., 1997) which is dominated by inflammation and eventually spreads across the entire neuroaxis (Park et al., 2004). We anticipate that remote cord atrophy therefore relates to neurodegenerative changes which are associated with chronic inflammation, retrograde degeneration of descending fibers and anterograde degeneration of ascending fibers (Beirowski et al., 2005; Freund et al., 2013; Grabher et al., 2015), as well as trans-synaptic changes within the propriospinal system (Filli and Schwab, 2015; Huber et al., 2018). Crucially, the magnitude of atrophy and microstructural changes at baseline were associated with sensorimotor recovery at post-SCI. This suggests that in addition to neurodegenerative changes, time-dependent reorganization of neuronal circuits may explain the association between SCI-induced atrophy and motor and sensory function (Courtine et al., 2008; Jain et al., 2000; Lundell et al., 2011).

At the level of the cerebellum, cerebellar circuit plasticity in response to injuries of the spinal cord have been reported to affect the integrity of ascending spinocerebellar pathways (Visavadiya and Springer, 2016). Interestingly, we found volumetric decreases. Next to atrophic changes we found R2* and MT parameter changes. R2* is associated with iron content (Langkammer et al., 2010), a key co-factor in the production and maintenance of myelin (Zecca et al., 2004). On the other hand, MT values have been associated with myelin content (Schmierer et al., 2007a, Schmierer et al., 2004) and thus this finding in the cerebellum may be indicative of myelin breakdown triggered by oxidative stress leading to release of iron.

Crucially, less atrophy in the cerebellum was associated with better sensory outcomes and increases in R2* in the cerebellum was associated with lower extremity motor scores. The association between structural changes in the cerebellum with sensorimotor outcomes highlights the role of the cerebellum in recovery processes after SCI (Grabher et al., 2015; Kaushal et al., 2016). The observed changes might serve to facilitate recruitment of neural substrates to compensate for neural deficits following SCI.

Further upstream, cortical thinning occurred within the leg representing area of M1 and S1 at baseline. This reduction in cortical thickness has been linked to soma size shrinkage of pyramidal cells and it is likely to be of permanent nature as this reduction persists into the very chronic state (Freund et al., 2011). In addition, diffusivity changes in the corticospinal tract (Freund et al., 2012) and myelin changes in M1 (Ziegler et al., 2018) become evident, demonstrating the sensorimotor system is affected in its entire length after SCI.

Finally, GM atrophy was evident within the limbic system. Specifically, we found volumetric decreases in the thalamus, anterior insula, inferior frontal gyrus, and anterior cingulate gyrus at baseline; most of which are in line with previous studies (Chen et al., 2017; Grabher et al., 2015; Jutzeler et al., 2016). These regions are relatively heterogeneous in terms of their function and cytoarchitecture. For instance, they are engaged in emotional function, depression, bladder controls, and cognitive control (Etkin et al., 2011; Griffiths et al., 2007; Tops and Boksem, 2011) next to the sensorimotor functions. However, alterations in structure and function in most of these areas have been associated with impaired sensorimotor processing in patients with SCI (Grabher et al., 2015). Interestingly, the progression of atrophy is paralleled by iron accumulation within the thalamus (Ziegler et al., 2018) indicating ongoing pathophysiological processes which accumulate over time and are associated with the development of neuropathic pain after SCI (Gustin et al., 2014; Jutzeler et al., 2016).

In summary, our results next to the former reports of dynamic structural brain changes in sub-acute patients (Chen et al., 2017; Hou et al., 2014; Jurkiewicz et al., 2007) show that the majority of changes within the brain are initiated very early following injury and are independent of lesion severity and level.

This study had a number of limitations. Patients were on average 10 years older than controls that may affect compensatory effects. However, age was considered as a covariate in all statistical analyses to exclude any age-related effects. The determined MR parameters (MT, R1, and R2*) are an indirect measure of macromolecular contents in tissue structure. We therefore cannot exclude a partial contribution of unexplored physiologic/cellular processes occurring after SCI. Although, previous reports have shown a high correspondence between MT-based measures and myelin staining in ex-vivo studies (Schmierer et al., 2004), as well as a dominate contribution of macromolecular component on R1 (Grabher et al., 2015; Rooney et al., 2007; Schmierer et al., 2004). Additionally, a post-mortem study showed the relationship between R2* and chemically determined iron concentrations (Langkammer et al., 2010). The acquisition time of MPM research protocol is relatively long for its clinical application; however, within our next studies we succeeded to reduce the acquisition time to 18 min.

In conclusion, remote macro- and microstructural neurodegenerative changes at the level of the spinal cord and brain occur at the very early stage following SCI, although with different temporal and spatial dynamics. Crucially, both early cord and cerebellar changes predict functional recovery independently of early clinical status. These findings provide new insights into the neurodegenerative mechanisms following SCI and begin to identify biomarkers which may predict the evolution of individual patients.

## Study funding

This study is funded by the SRH Holding, Wings for Life Austria (WFL-CH-007/14), the EU project (Horizon 2020 ‘NISCI’ grant agreement n_ 681094), ERA-NET Neuron (hMRI of SCI) and the Clinical Research Priority Program “NeuroRehab” of the University of Zurich.

## Author contributions

MS: data analysis, statistical analysis, conception and design of the study, drafting and finalizing the manuscript; AC, AT, NW: conception and design of the study; PG: data acquisition, drafting manuscript; PF: conception and design of the study, drafting and revising the manuscript.

## Conflicts of interest

We declare no conflicts of interest.

## Disclosure statement

The Wellcome Trust Centre for Neuroimaging and Max Planck institute in Germany have an institutional research agreement with Siemens Healthcare and receive support from Siemens. Prof. Alan J. Thompson has received honoraria and support for travelling and consultancy from Biogen Idec, MedDay, Eisai, and Novartis, and for teaching from Teva, Novartis, and EXCEMED. He also receives an honorarium as editor-in-chief of Multiple Sclerosis.
